# A phase II, double blind, placebo-controlled, randomized evaluation of the safety and efficacy of tafenoquine in patients with mild-moderate COVID-19 disease

**DOI:** 10.1016/j.nmni.2022.100986

**Published:** 2022-06-01

**Authors:** G.-S. Dow, B.-L. Smith

**Affiliations:** 60 Degrees Pharmaceuticals LLC, Washington DC, 20036, United States

**Keywords:** Tafenoquine, COVID-19, Outpatients, Clinical Trial, FDA-Approved Regimen for Malaria Pophylaxis

## Abstract

The safety and efficacy of tafenoquine administered as a 200 mg dose once per day on days 1, 2, 3, and 10 was evaluated over a 28-day period in mild-moderate COVID-19 patients. The primary endpoint was Day 14 clinical recovery from COVID-19 symptoms, defined as cough mild or absent, respiratory rate < 24 bpm, and no shortness of breath or fever. Following a successful futility analysis after n = 86 patients out of a target n = 275 were randomized, the study was terminated and unblinded early to facilitate planning for confirmatory studies. The proportion of patients not recovered on Day 14 was numerically decreased by 27% in the ITT population [8/45 v 10/42 not recovered in the tafenoquine and placebo arms, *P* = 0.60] and 47% in the PP population [5/42 v 9/41, *P* = 0.25]. Amongst individuals who recorded responses in an electronic diary at Day 28, all tafenoquine patients were recovered, whereas up to 12% of placebo patients exhibited lingering dyspnea. Time to clinical recovery from COVID-19 symptoms was accelerated in the tafenoquine arm by about 2-2.5 days. There were two COVID-19 related hospitalizations in the placebo arm and one in the tafenoquine arm. Mild, drug related adverse events occurred in 8.4% of individuals in the tafenoquine arm [v 2.4% in the placebo]. Although this trial was underpowered for the primary endpoint due to its early termination, the data are suggestive of a therapeutic benefit associated with tafenoquine administration in outpatients with mild to moderate COVID-19 disease, and larger studies are planned.

## Introduction

Vaccination is an effective strategy for reducing the risk of severe COVID-19 disease and blunting community transmission of SARS-CoV-2 [1]. However, breakthrough infections occur frequently, and, prior to emergence of omicron variant, represented approximately 17% of disease burden in the United States [2]. At the time this study was planned, clinical trial results for fluvoxamine, molnupiravir and paxlovid had not been reported, and, in any case, these drugs have not been evaluated in vaccinated patients or in milder disease with low risk of disease progression.

Tafenoquine is approved in the United States, Australia, Brazil, Peru and Thailand for treatment of *P. vivax* malaria [300 mg dose] or for malaria prophylaxis in non-immune travelers [loading dose of 600 mg over three days followed by weekly administration of 200 mg] [[3], [4], [5], [6]]. The prophylaxis regimen was recently shown to have a similar adverse event profile to placebo over 12-month period of continuous dosing healthy volunteers [7]. The weekly dosing and long half-life of tafenoquine [16 days] may offer the possibility of more convenient dosing regimens than other oral COVID-19 therapeutics that have completed Phase III clinical trials.

Tafenoquine exhibited an EC50/90s against SARS-CoV-2 of 2.6/5.1 μM and 8.6/17 μM in VERO and human epithelial cells, respectively, and was at least a thousand-fold more potent than other aminoquinoline antimalarials when accounting for protein binding [8,9]. Although a weak-base like mechanism in VERO cells cannot be ruled out, inhibition of viral and host proteases is the suspected mechanism [10,11]. Pharmacokinetic simulations of the approved prophylactic dose showed that free intracellular concentrations of tafenoquine in the lung exceed antiviral EC90s for at least three weeks [8,9]. This scientific rationale facilitated granting of a “clearance to proceed” letter from the FDA for this study.

The objective of the clinical study described herein was to assess whether the first four doses of the FDA-approved malaria prophylactic regimen of tafenoquine [200 mg once per day for three days, then a single dose of 200 mg one week later for a total dose of 800 mg] improved clinical recovery [assessed at Study Day 14] from COVID-19 symptoms in ambulatory patients. The study also evaluated the effect of tafenoquine on other efficacy [e.g., time to clinical recovery] and safety endpoints.

## Methods

### Ethics and informed consent

This study was conducted under FDA jurisdiction [IND # 152,009]. The study protocol, consent forms, and patient information sheets were approved by a central institutional review board [Advarra, Inc] and the U.S Department of Defence's Human Research Protection Office [HRPO] prior to the initiation of study activities, and all amendments/addenda to the protocol were approved by these review boards prior to their implementation. Study patients were fully informed of the nature of the study, the properties and side effects of tafenoquine, and all relevant aspects of study procedures in the informed consent document and during recruitment. Also, patients could ask questions of study personnel at any time during the trial.

### Study Sites and Patient Eligibility

This was a randomized, double-blind, placebo-controlled trial in eighty-six patients with mild-moderate COVID-19 disease, conducted at 15 outpatient sites across the United States, enrolled between March 1st, 2021, and September 10th, 2021, and managed by Peachtree Bioresearch Solutions [see list of acknowledged sites, principal investigators and CRO staff in the Acknowledgements]. Patients randomly received either tafenoquine or placebo in a 1:1 ratio. Eligible patients had PCR-confirmed SARS-CoV-2 infection [any U.S. FDA approved laboratory test], any of the following COVID-19 symptoms within 5 days of and inclusive of screening: respiratory rate > 24 bpm on room air, new cough or shortness of breath or fever [temperature ≥ 37.7°C], normal G6PD enzyme activity levels, were able to be prescribed tafenoquine according to U.S. prescribing information for ARAKODA® for malaria prophylaxis, and did not have symptoms of longer than 7 days duration when the first dose of study medication was administered. Females of childbearing potential agreed to either true sexual abstinence or to use acceptable contraceptive methods. Eligible patients would also have to comply with all study-related visits/procedures over the full trial period and to remain in contact with the study site throughout the trial. Patients were excluded if they exhibited signs of severe COVID-19 disease or symptoms consistent with imminent hospitalization [within 48h], any significant medical issues in the last seven days, were pregnant, breast-feeding or had taken/planned to take COVID-19 therapeutics within 30 days/during the study. Prior COVID-19 vaccination was not exclusionary.

### Randomization and blinding

Administration of tafenoquine or placebo was block randomized within sites and stratified across sites using randomization codes generated by an interactive web response system [IWRS]. The following parties were blinded to identity of study drug: Investigators, patients, laboratories, sponsor, and study team [including data management, statistician, and programmers]. Only the IWRS and randomization statistician were aware of treatment assignment.

### Tafenoquine administration, study visits, sample collection, and follow-up

Tafenoquine [or matching placebo manufactured for 60P by Piramal Enterprises Limited] was administered orally as 2 × 100 mg tablets on study days 1, 2, 3, and 10. The first dose was administered in the study clinic [Day 1] and was directly observed [this was to ensure drug was not taken until after normal G6PD status was confirmed] and to document symptoms at baseline [i.e. prior to the first dose]. The remaining tablets were provided to patients in a child-proof HDPE plastic container to self-administer at home. Patients were asked to report the severity of 14 COVID-19 symptoms using a self-assessment scale [see Supplementary Table 1] at Screening, and from Day 1 through Day 28, with Day 1 and 14 responses being recorded as clinic visits and the remainder remotely using an online web-based application. At the same times [with the exception of the screening visit], patients were asked to self-report adverse events. These were verified by study staff in daily telemedicine visits [unless the daily visit was a clinic visit]. Medical history was collected during screening and baseline visits. Blood samples for hematology and clinical chemistry were collected at screening [baseline] and at the Day 14 clinic visit. The primary endpoint (clinical recovery) was assessed at the Day 14 clinic visit.

### Endpoints and analysis populations

The primary study endpoint was clinical recovery at Day 14, defined as no fever [oral or skin temperature ≤ 37.7° C], cough reported by the patient as mild or absent, no shortness of breath as reported by the patient, and respiratory rate ≤ 24 breaths per minute [bpm] on room air. Analysis populations were all randomized patients [intent-to-treat, ITT], all randomized patients who completed the Day 14 in clinic visit [per protocol, PP] and ITT patients who took at least one dose of study medication [safety population]. Patients with major protocol deviations were not excluded from the PP population.

Safety analyses included adverse events, vital signs, hematology and clinical chemistries. Secondary endpoints included patient reported COVID-19 symptoms on Days 14, incidence of hospitalization and number of medical follow up visits. Exploratory efficacy analyses included proportion clinically recovered on Day 28, time to clinical recovery for the main study endpoint, and time to maximum severity for the four components of the primary study endpoint.

Post-hoc analyses included proportion recovered on Day 28 amongst the ITT and PP populations for whom a Day 28 response was recorded, aggregate patient reported symptom scores across 14 COVID-19 symptoms from Days 1–28, a sensitivity analysis was conducted post-hoc for the primary endpoint for the PP population, by removing those patients with major protocol deviations, and clinical recovery curves for the individual symptoms comprising the primary endpoint. In the latter analysis, patients were included if they exhibited the specified primary endpoint symptom during the first three days of tafenoquine/placebo administration [Days 1–3] and were assessed as recovered the first time they had not experienced the symptoms for three consecutive days after completion of the first three days of dosing.

### Adverse event and anomalous lab value characterization

Adverse events were characterized based on severity [mild, moderate, severe], relatedness to study drug [definitely, probably, possibly, unlikely, unrelated], as a serious adverse event [SAE] if they involved hospitalization or death or as an unanticipated problem involving risk to subjects or others [UPIRTSO]. Adverse events definitely, probably, and possibly related are reported as drug-related whereas adverse events recorded as unlikely or unrelated are reported herein as unrelated to study medications. The 14 self-reported COVID-19 symptoms were not considered to be adverse events [this was protocol-defined]. Hospitalizations for COVID-19 symptoms by protocol were expected outcomes given the disease in question.

### Statistical analysis

Logistic regression model was used to analyze the primary endpoint, with the following covariates of interest defined in the protocol/statistical analysis plan (SAP) prior to unblinding: Age [<40 or ≥40 years old], vaccination [any prior vaccination or no prior vaccinations] duration of COVID-19 symptoms prior to screening [≤ 2 v > 2 days], and any medical history of cardiovascular or metabolic disease. The sample size of the study [N = 275, to achieve 250 completers assuming 10% loss to follow-up] was selected such that the study was powered to detect a difference of 30% not recovered in the placebo arm versus 15% not recovered in the tafenoquine arm, with 80% power and two-sided alpha of 0.05. Other than the exceptions outlined in the next paragraph, pre-planned efficacy and safety outcomes described herein were analyzed in accordance with the SAP.

There were three general exceptions to the above: [i] the study was terminated early for business [not safety] reasons, as outlined elsewhere in this report, [ii] the per protocol analysis population included subjects with major deviations, and [iii] several exploratory post-hoc analyses were performed as described earlier. With respect to the second point, the original SAP called for subjects with major deviations to be excluded from the PP population—prior to unblinding, the sponsor elected not to exclude subjects with major deviations from the PP population.

### Futility Analysis and early termination

During the course of the study, a protocol mandated futility analysis was conducted when N = 86 of an intended N = 275 [12]. The study's data safety monitoring board [DSMB] recommended completion of enrolment without protocol modification [13], after it was determined that the conditional probability of achieving statistical significance for the main study endpoint exceeded the priori-determined threshold of 43%. This threshold was set by the sponsor at this level because the attrition for infectious disease products in Phase II clinical programs is 43% [14]. At that point, the sponsor elected, for business reasons articulated later in this report, to terminate and unblind the study early [15].

## Results

### Patient demographics and flow chart

**Fig. 1:**
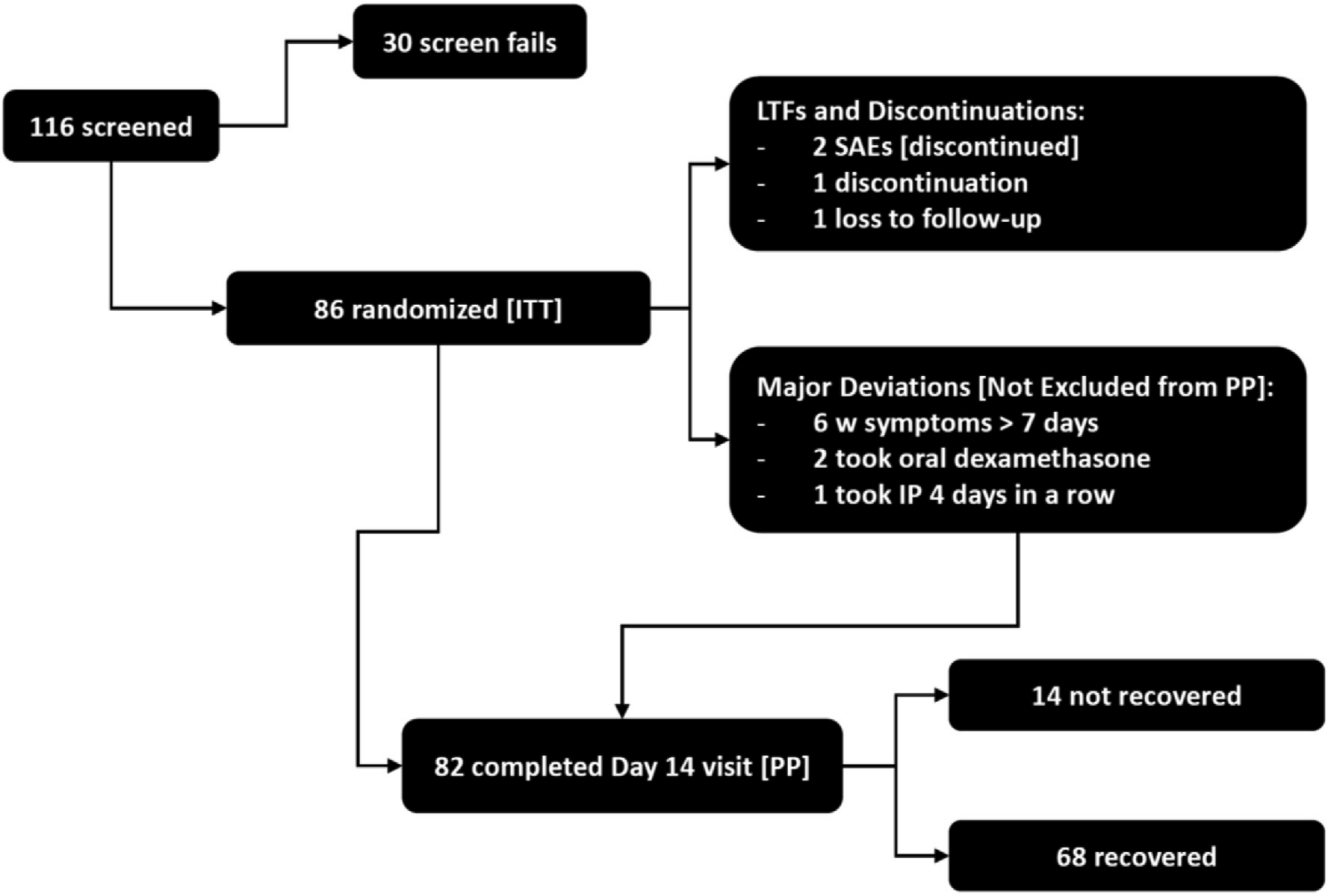
Study flowchart.

The tafenoquine and placebo groups were balanced across all demographics and covariates except that the proportion of individuals with shortness of breath was higher in the placebo arm [Table 1]. However, the composite primary endpoint, and aggregate patient reported COVID-19 symptoms were balanced across the groups. There were 45 and 41 randomized patients in the tafenoquine and placebo arms [ITT population, see Fig. 1] and all patients took at least one dose of study medication [safety population]. Compliance with study medication was similar between the groups. The four individuals excluded from the PP analysis included two COVID-19 hospitalizations [one tafenoquine, one placebo], one discontinuation due to withdrawal of consent [tafenoquine] and one loss to follow-up [placebo]. The third hospitalized patient [placebo] was withdrawn from medication when hospitalized, continued in the study inclusive of the Day 14 visit, and was therefore not excluded from the PP population. There were nine major deviations: Six were for patients administered study drug after the seven-day symptom window [four tafenoquine, two placebos], two were for patients who took oral dexamethasone [both placebo] and one patient took IP 4 days in a row [tafenoquine, see Fig. 1].

**Table 1:** Patient demographics and baseline symptoms

Parameter	Tafenoquine [n = 45]	Placebo [N = 41]
Demographics		
Female [N, (%)]	24 (53%)	21 (51%)
Age [years ± SD]	43 (15)	43 (15)
Height [cm ± SD]	168 (11)	167 (11)
Weight [kg +/SD]	83 (14)	85 (23)
Race: White [%]	43 (96)	41 (100)
Ethnicity: Hispanic or Latino [%]	35 (78)	30 (73)
Major Deviations		
Symptom duration > 7 days on Day 1 [N, (%)]	4 (8.9%)	2 (4.9%)
Patient took oral dexamethasone [N, (%)]	0 (0%)	2 (4.9%)
Patient took study medication 4 days in a row	1 (2.4%)	0 (0%)
Covariates		
Age > 40 [N (%)]	25 (56%)	24 (59%)
Vaccinated [N (%)]	14 (31%)	14 (34%)
Duration of symptoms < 2 days prior to screening	27 (60%)	25 (61%)
Cardiovascular or metabolic disease	17% overall, not included in covariate analysis	
Primary outcome symptoms on Day 1		
Fever [N, (%)]	4 (8.9%)	5 (12%)
Shortness of breath [N, (%)]	17 (38%)	25 (61%)
Respiratory rate > 24 bpm (N, (%)]	0 (0%)	0 (0%)
Cough moderate or severe [N (%)]	25 (56%)	23 (56%)
None of the above recorded on Day 1 [N (%)]^a^	14 (31%)	9 (22%)
14 Patient Reported COVID-19 Symptoms		
Aggregate group score	575	544
Mean [SD]	12.77 [6.3]	13.27 [5.4]

a Recorded as recovered from all symptoms or missing data made assessing recovery impossible.

### Clinical recovery at day 14

Tafenoquine numerically reduced the proportion of individuals not clinically recovered on Day 14 in the ITT and PP populations by 27% and 47% without reaching the level of statistical significance [Table 2]. In a sensitivity analysis, it was determined that exclusion of patients from the PP population resulted in a small decrease [to 44%] in the proportion of patients not clinically recovered on Day 14 [Table 3]. Covariate analysis was conducted on the primary endpoint [clinical recovery on Day 14]. None of the covariates reached the level of statistical significance. Prior vaccination exhibits a trend towards improved clinical recovery in both the ITT and PP populations, which was more pronounced than either of the other non-treatment covariates. In the PP population, the impact of tafenoquine and prior vaccination were similar [OR 2.2 and 2.4, respectively, see Table 2].

**Table 2:** Pre-planned efficacy outcomes

Population	Tafenoquine (TQ)	Placebo (PL)
Day 14 Clinical Recovery [ITT]		
Randomized patients [N], patients not recovered [N, %]	45, 8 (17.8%)	41, 10 (24.4%)
Patients recovered [N, (%, 95% CI)]	37 (82.2%, 68–92%)	31 (75.6%, 60–88%)
Change in proportion unrecovered in TQ v PL, P	27%, 0.60	
OR treatment [TQ]	1.56 (0.54–4.5)	
OR age [< 40 years]	1.47 (0.46–4.4)	
OR vaccination [yes]	3.01 (0.74–12)	
OR symptoms prior to enrollment [≤ 2 days]	1.36 (0.45–4.1)	
Day 14 clinical recovery [PP]		
Randomized patients [N], patients not recovered [N, %]	42, 5 (11.9%)	40, 9 (22.5%)
Patients recovered [N, (%, 95% CI)]	37 (88.1%, 74%–96%)	31 (77.5%, 62%–89%)
Change in proportion unrecovered in TQ v PL, P	47%, 0.25	
OR treatment [TQ]	2.2 (0.67–7.5)	
OR age [< 40 years]	1.3 (0.38–4.5)	
OR vaccination [yes]	2.5 (0.58–11)	
OR symptoms prior to enrollment [≤ 2 days]	0.78 (0.21–2.8)	
Day 28 clinical recovery [ITT]		
Randomized patients [N], patients not recovered [N, %]	45, 7 (15.5%)	41, 9 (22%)
Patients recovered [N, (%, 95% CI)]	38 (84%, 71%–94%)	32 (78%, 62%–89%)
Change in proportion unrecovered in TQ v PL, P	30%, 0.58	
Day 28 clinical recovery [PP]		
Randomized patients [N], patients not recovered [N, %]	42, 4 (9.5%)	
Patients recovered [N, (%, 95% CI)]	38 (90%, 77%–97%)	40, 8 (20%)
Change in proportion unrecovered in TQ v PL, P	58%, 0.22	32 (80%, 64%–91%)
ITT hospitalizations due to COVID-19 symptoms		
Randomized patients N, patients hospitalized [N, %]	45, 1 (2.2%)	41, 2 (4.9%)
Decrease in proportion hospitalized in TQ arm, P	55%, 0.60	
ITT time to clinical recovery: N, mean (SE)	45, 4.4 (0.69)	41, 6.5 (0.84)
*P* no covariates/*P* covariates	0.05/0.11	
PP time to clinical recovery: N, mean (SE)		
*P* no covariates/*P* covariates	42, 4.0 (0.62)	40, 6.3 (0.84)
[data for other primary endpoint symptoms not shown]	0.02/0.07	
Time to maximum severity, fever: N, mean (SE)	45, 4.5 (0.77)	41, 7.5 (1.4)
*P* no covariates, *P* covariates	0.03/0.09	
Patient reported severity of 14 COVID-19 symptoms on Day 14 [ITT]		
Stuffy or runny nose [none]	35 (78)	31 (76)
Sore throat [none]	39 (87)	37 (90)
Shortness of breath [none]	38 (84)	32 (78)
Cough [none]	34 (76)	25 (61)
Low energy or tiredness [none]	31 (69)	21 (51)
Muscle of body aches [none]	34 (76)	33 (81)
Headache [none]	42 (93)	39 (95)
Chills or shivering [none]	41 (91)	39 (95)
Feeling hot or feverish [none]	41 (91)	39 (95)
Nausea [none]	41 (91)	39 (95)
Vomiting [none in last 24 h]	42 (93)	39 (95)
Diarrhea [none in last 24 h]	39 (87)	37 (90)
Sense of smell [same as usual]	19 (42)	17 (41)
Sense of taste [same as usual]	20 (44)	15 (37)

**Table 3:** Exploratory post-hoc analyses

Population	Tafenoquine (TQ)	Placebo (PL)
Day 28 Clinical recovery amongst patients who responded [ITT]		
Randomized patients [N], patients not recovered [N, %]	41, 3 (7.3%)	
Patients recovered [N, (%, 95% CI)]	38 (93%, 80%–99%)	39, 7 (18%)
Change in proportion unrecovered in TQ v PL, *P*	59%, 0.19	32 (82%, 67%–93%)
Day 28 Clinical recovery amongst patients who responded [PP]		
Randomized patients [N], patients not recovered [N, %]	38, 0 (0%)	
Patients recovered [N, (%, 95% CI)]	38 (100%, 91%–100%)	39, 7 (18%)
Change in proportion unrecovered in TQ v PL, *P*	100%, 0.012	32 (82%, 67%–93%)
Day 14 clinical recovery [PP with patients with major deviations excluded]		
Randomized patients [N], patients not recovered [N, %]		
Patients recovered [N, (%, 95% CI)]	37, 4 (11%)	
Change in proportion unrecovered in TQ v PL, *P*	33 (89%, 75%–97%)	36, 7 (19%)
	44%, 0.34	29 (81%, 64%–92%)

### Clinical recovery at day 28

In the pre-planned clinical recovery at Day 28 endpoints, tafenoquine reduced the proportion unrecovered by 30 and 58% in the ITT and PP populations [Table 2]. However, since there were no actual clinically unrecovered patients amongst Day 28 respondents in the tafenoquine arm, post-hoc analyses were conducted for the PP and ITT populations who completed the Day 28 survey [Table 3]. Tafenoquine reduced the proportion unrecovered in the ITT and PP populations by 59% and 100% [Table 2], with *P* = 0.012 for the latter observation.

Of the seven tafenoquine patients for whom a Day 28 visit was not recorded, three left the study prior to Day 14 (1 loss to follow-up, 1 hospitalization, 1 withdrawal of consent), two were lost to follow up after Day 14 after recovering, and two were lost to follow up having recorded shortness of breath in their last diary entry. Of the 9 placebo patients in the ITT population not listed as recovered on Day 28, 5 were recorded as having due to shortness of breath, two had missing respiratory rate data so recovery/not recovery could not be determined but were otherwise recovered, and two were hospitalized so a Day 28 outcome was not recorded.

### Time to clinical recovery [For symptoms comprising the primary endpoint]

The time to clinical recovery was examined in both ITT and PP populations. In the ITT population, tafenoquine numerically shortened the average time to clinical recovery by 2.1 days on average [4.4 (SE 0.69) days v 6.5 (SE 0.84) days for placebo, *P* = 0.053, Table 2]. In the PP population, tafenoquine numerically shortened the average time to clinical recovery by 2.3 days [4.0 (SE = 0.64) days v 6.3 (SE = 0.84) days for placebo, *P* = 0.02, Table 2]. The time to maximum severity amongst the primary endpoint symptoms was shorter for tafenoquine than placebo for fever [Table 2] but not the other symptoms [data not shown].

**Fig. 2:**
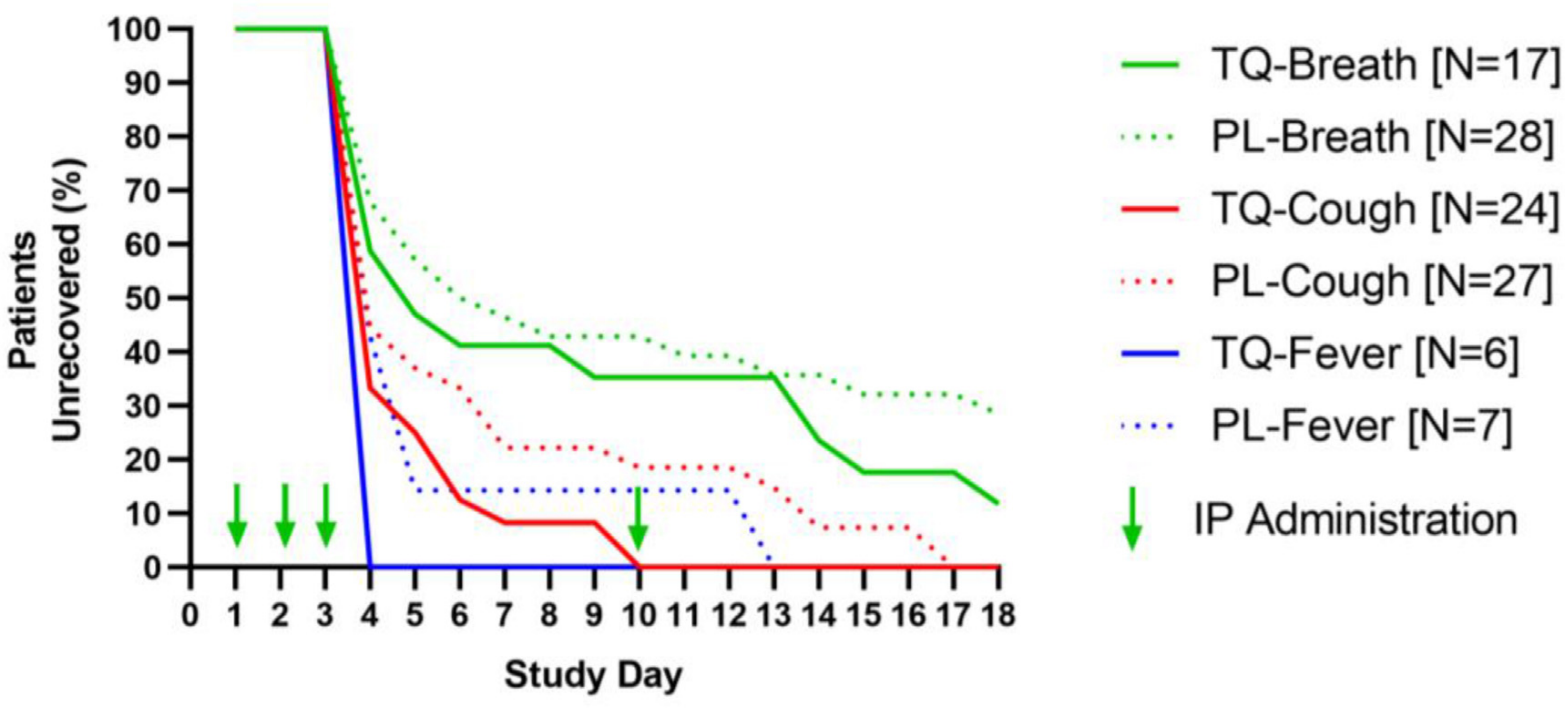
Recovery from symptoms comprising the composite primary endpoint in the PP population. Patients were included if they experience symptoms on any of Days 1-3 and considered recovered if the symptom was resolved for three consecutive days. IP refers to investigational product [tafenoquine or placebo]. The figure is truncated at Day 18 since only placebo subjects continued to experience symptoms through Day 28 [shortness of breath]. The two tafenoquine subjects experiencing shortness of breath at Day 18 were lost to follow-up thereafter. When all components of the primary endpoint were considered together, tafenoquine patients recovered 2.3 days faster than placebo patients in the PP population [*P* = 0.02, see Table 2].

Since shortness of breath was more common in the placebo group than the tafenoquine group at baseline, recovery curves for shortness of breath, fever, and cough were evaluated qualitatively in post-hoc analyses. Recovery from each of these symptoms appeared to be numerically faster in the tafenoquine group [Fig. 2], and all patients recovered from fever and cough prior to Day 18 in both groups. At Day 18 there were numerically fewer tafenoquine patients with lingering shortness of breath compared to placebo [2 v 8]. The two tafenoquine subjects were lost to follow up, and shortness of breath lingered in seven of the placebo subjects through Day 28 [data not shown]. Note that since this analysis included patients with shortness of breath on any of Days 1, 2, or 3, and had a more stringent definition of recovery, the number of patients [n = 7] in the placebo arm with shortness of breath at Day 28 is different than the number for the whole cohort [n = 5].

### Time to clinical recovery [Patient reported outcomes]

**Fig. 3:**
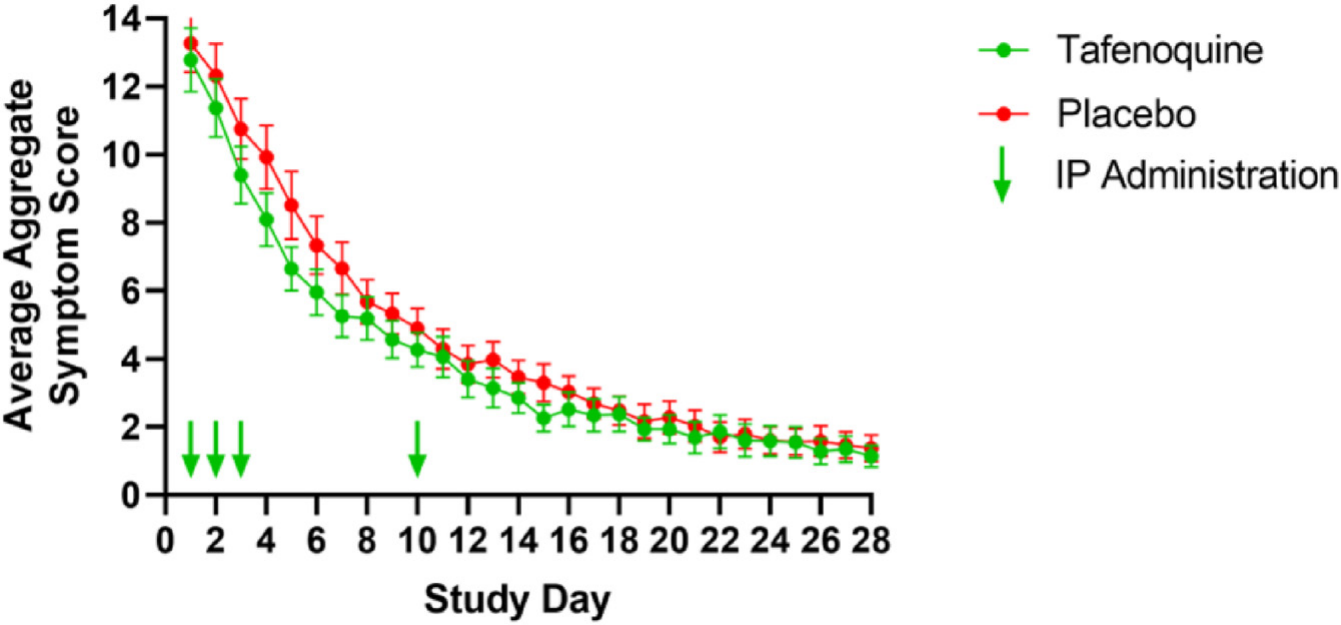
Effect of tafenoquine on 14 patient-reported COVID-19 symptoms in the ITT population. IP = investigational product. The lowest *P* value, on Day 5, was 0.059.

The proportion of patients reporting as feeling normal across 14 patient-reported symptoms was numerically similar to or higher in tafenoquine patients than in placebo patients on Day 14 [Table 2]. This observation and the pre-planned analysis of time to clinical recovery led to an exploratory post-hoc longitudinal analysis of average aggregate patient reported outcomes. As is evident from Fig. 3, the time taken for a 50% improvement in symptom scores was about two days shorter in the tafenoquine v placebo arms. The greatest difference between tafenoquine and placebo occurred on Day 5 and was associated with a *P*-value of 0.059 on Day 5.

### Safety, adverse events, and COVID-19 hospitalizations

There were three SAEs, all COVID-19 hospitalizations, one in the tafenoquine arm, and two in the placebo arm—none of these were UPIRTSO or required expedited reporting to the FDA [Table 2, Table 4]. All three hospitalized individuals recovered and were discharged. Six mild drug-related events were observed in four (8.9%) tafenoquine patients whereas one placebo (2.4%) patient had two mild-moderate adverse events. There were five mild-moderate adverse events unrelated to drug product amongst four patients in the tafenoquine arm and eleven mild-severe adverse events unrelated to drug amongst three patients in the placebo arm. The incidences and severities of individual adverse events are reported in Table 4. Drug was discontinued in one patient while elevated transaminase levels were investigated, and in the three patients hospitalized with COVID-19 symptoms [Table 4].

**Table 4:** Adverse events

MedRA term & relatedness	Incidence [N, (%)]						
	Tafenoquine (N = 45)			Placebo (N = 41)			
	Mild	Moderate	SAE	Mild	Moderate	Severe	SAE
Related to study medication							
Diarrhea	1 (2.2%)	—	—	1 (2.4%)	—	—	—
Hematocrit decreased	1 (2.2%)	—	—	—	—	—	—
Hemoglobin decreased	1 (2.2%)	—	—	—	—	—	—
Hypotension	1 (2.2%)	—	—	—	—	—	—
Rash	1 (2.2%)	—	—	—	—	—	—
Red blood cell count decreased	1 (2.2%)	—	—	—	—	—	—
Vomiting	1 (2.2%)	—	—	—	1 (2.4%)	—	—
Not related to study medication							
Abdominal upper pain	—	—	—	1 (2.4%)	—	—	—
Alanine amino transferase increased	—	—	—	1 (2.4%)	—	—	—
Bradycardia	—	—	—	1 (2.4%)	—	—	—
Bronchitis	1 (2.2%)	—	—	—	—	—	—
COVID-19 pneumonia	—	1 (2.2%)^a^	1 (2.2%)^a^	—	—	2 (4.9%)^a^	2 (4.9%)^a^
Dizziness	—	—	—	1 (2.4%)	—	—	—
Dyspnea	—	—	—	—	1 (2.4%)	—	—
Epistaxis	—	—	—	1 (2.4%)	—	—	—
Hypertension	—	—	—	1 (2.4%)	—	—	—
Panic attack	—	1 (2.2%)	—	—	—	—	—
Sinus congestion	—	1 (2.2%)	—	—	—	—	—
Syncope	—	—	—	—	1 (2.4%)	—	—
Transaminase increased	—	1 (2.2%)^a^	—	1 (2.4%)	—	—	—

a AE led to study drug discontinuation/disruption of study continuity.

Except as otherwise noted in this paragraph, there were no clinically meaningful mean changes from baseline to Day 14, or in tafenoquine v placebo Day 14 group averages for standard vital signs, hematologic or clinical chemistry parameters. Numerical increases in creatinine between baseline and Day 14 were higher in the tafenoquine arm. Numerical decreases in hemoglobin and hematocrit were more pronounced in the tafenoquine arm. Mild decreases in hematocrit, hemoglobin, and red cell count were observed in one tafenoquine patient, which were not clinically significant. A transaminase elevation occurred in one tafenoquine patient but upon investigation was not found to be clinically significant.

## Discussion

At the time of the unblinding of this study, topline results from Phase III studies for three oral COVID-19 therapeutics in at risk patients with mild-moderate COVID-19 disease had been announced: Fluvoxamine, molnupiravir, and paxlovid reduced the risk of hospitalization by approximately 30%, 50% [in the first public announcement] and 90%, respectively [[16], [17], [18]]. The sponsor had conducted an interim analysis in which the DSMB recommended completion of enrolment but did not know the magnitude of the potential therapeutic benefit accruing to tafenoquine. An informal survey of potential funding/commercialization partners for a Phase III program suggested it would be important to know the magnitude of possible benefit prior to initiating such an effort. Primarily for the above reasons, the present study was terminated and unblinded early.

Based on the COVID-19 symptoms selected for primary endpoint, tafenoquine numerically reduced the incidence of individuals not clinically recovered on Day 14 by 27%–47% and on Day 28 by 30%–100%. These trends must be interpreted with caution since they did not reach the level of statistical significance. The major limitations of this study, that is, its small size and early termination, are the likely the reason for this. However, the data do not rule out that tafenoquine may exhibit a therapeutic effect in non-hospitalized COVID-19 patients of a similar order of magnitude to the aforementioned therapeutics.

One can infer from the recruitment date in this study, and the fact that most recruitment occurred between late July 2021 and mid-September 2021, that the majority of patients may have been infected by the delta variant of SARS-CoV-2. Therefore, it is not known whether the efficacy results of this study would be applicable to other variants, such as omicron, which became dominant after the study was terminated. This is a general limitation of all COVID-19 intervention studies, since the SARS-CoV-2 virus mutates so rapidly.

The large COVID-19 clinical trial platforms, have, or are contemplating, a shift from hospitalization to time to clinical recovery, as the PRINCIPLE platform in the UK has done for some of its interventional studies [19]. This is partly in anticipation of COVID-19 disease becoming milder with less severe illness and the disease burden shifting to include more vaccinated individuals, as execution of clinical trials focused on hospitalization endpoints will become more difficult and less relevant to the course of illness in the majority of individuals. The present study suggests that tafenoquine has the potential to decrease clinical recovery time, and the time to achieve a reduction in 50% of symptom burden across an array of COVID-19 symptoms, by about 2–2.5 days, trends that approached statistical significance. These observations should be confirmed in a larger study.

The incidence of drug-related events in the tafenoquine arm was similar to that reported for the same regimen in a malaria challenge study [20]. Small decreases in hematological parameters related to the red cell compartment were observed in that study, and in the recent long-term evaluation of the safety of tafenoquine over 12 months of continuous dosing [7]. During continuous weekly prophylaxis, these parameters return to baseline within about twelve weeks without discontinuation of drug [7]. The numerically larger changes in creatinine in the tafenoquine arm were also expected, have been observed in other studies including the long-term safety study, and are not clinically significant [7]. There was nothing in terms of the safety profile of tafenoquine in this study that would preclude execution of a larger confirmatory study.

Assuming clinical benefit is confirmed in a larger study, tafenoquine has potential advantages and disadvantages over other oral COVID-19 therapeutics. Tafenoquine administration requires administration 200 mg [as 2 × 100 mg tablets] once per day on three days [total 6 tablets] then 2 × 100 mg tablets once weekly, whereas fluvoxamine, molnupiravir, and paxlovid have 20-, 40-, and 30-count pill burdens, respectively over 5–10 days and must be dosed twice daily. The demonstrated 12-month safety profile [7] distinguishes tafenoquine from molnupiravir and paxlovid, suggesting the possibility of pre-exposure prophylaxis or pre-treatment loading indications [for example, during travel of > 5 days duration]. In a travel medicine context, maintenance dosing of tafenoquine would be once weekly rather than twice daily. The main disadvantage of tafenoquine, reflected in the two-day delay between screening [up to five days of symptoms] and IP administration [no more than seven days of symptoms] in this study, is the requirement for a G6PD test prior to administration, usually performed by commercial pathology labs in most out-patient settings. In the Unites States, it is anticipated this limitation will be addressable through an increase in scale [tests are currently low volume so are batched for routine screening by commercial pathology labs] or point of care tests that can be performed in a primary care setting [as is the case in Australia].

The scientific rationale for this study was that tafenoquine acts as an antiviral, since pharmacokinetic modeling suggested the dose evaluated herein exceeded the EC90 against SARS-CoV-2 *in vitro* for at least three weeks [8,9]. The rapid clinical recovery in the tafenoquine arm beginning after two days of dosing could be consistent with either immunomodulatory or antiviral effects or both. The analysis of viral load and cytokine samples collected during the study are pending and may confirm whether tafenoquine exhibits an antiviral effect. We will report those data in a subsequent communication.

## Conclusions

In this small Phase II learning study, patients with mild-moderate COVID-19 disease who received tafenoquine for four days (200 mg on days 1,2,3, &10) appeared to recover more rapidly (by about two days), with a smaller proportion unrecovered on Days 14 and 28 (at least 27%), than those receiving placebo. Larger studies are being planned to confirm these observations.

## Funding sources

This project was funded jointly by 60 Degrees Pharmaceuticals and the U.S. Department of Defense’s (DOD) Joint Program Executive Office for Chemical, Biological, Radiological and Nuclear Defense (JPEO-CBRND) in support of the Defense Health Agency (DHA) under an Other Transaction Authority (OTA) agreement [Contract: W911QY2190011].

## Competing interests

GSD is the compensated CEO and CSO of 60P, the majority shareholder of 60P, an inventor on US patents 10342791 and 10,888,558 and US patent application 17/189544 [and related patents] and has a financial interest in the commercial success of Tafenoquine. BS is an inventor on US patents 10342791 and 10,888,558 and was paid in his capacity as the Chief Medical Officer of 60 Degrees Pharmaceuticals in relation to participation in this study. These statements are made in the interest of full disclosure and not because the authors believe these statements to constitute a conflict of interest.

## Credit statement

Geoffrey Dow: Methodology, Formal analysis, Project Administration, Resources, Supervision, Visualization, Writing – Original Draft Preparation.

Bryan Smith: Conceptualization, Formal analysis, Methodology, Supervision, Writing – Review and Editing.

## Consent to publish

The US Army and 60 Degrees Pharmaceuticals consented to publication of the material contained herein. The opinions or assertions contained herein are the private views of these authors and are not to be construed as official or reflecting the views of the U.S. Army or the U.S. DoD.
