# Supplementary material for: A phase II, double blind, placebo-controlled, randomized evaluation of the safety and efficacy of tafenoquine in patients with mild-moderate COVID-19 disease
Source: New Microbes New Infect. 2022 Jun 1;47:100986. doi: 10.1016/j.nmni.2022.100986 (PMC9158239; doi:10.1016/j.nmni.2022.100986)
Supplement: Multimedia component 1 [file mmc1.pdf]

Supplementary Table 1

The *FDA 2020 Guidance on Assessing COVID-19 Symptoms* 14 Common COVID-19 Symptoms Severity Scale will be used and is shown below. These patient-reported outcomes (PRO) was assessed by the patient and responses were recorded in the eDiary application. The score values are for analytical purposes and were not presented to patients in the diary.

Assessment of 14 Common COVID-19-Related Symptoms: Items, Response Option, and Severity Score

Item	Response Option and Severity Score
1. Stuffy or runny nose	None = 0<br>Mild = 1<br>Moderate = 2<br>Severe = 3
2. Sore throat	
3. Shortness of breath (difficulty breathing)	
4. Cough	
5. Low energy or tiredness	
6. Muscle or body aches	
7. Headache	
8. Chills or shivering	
9. Feeling hot or feverish	
10. Nausea (feeling like you wanted to throw up)	
11. How many times did you **vomit (throw up)** in the last **24 hours**?	I did not vomit at all = 0<br>1–2 times = 1<br>3–4 times = 2<br>5 or more times = 3
12. How many times did you have **diarrhea (loose or watery stools)** in the last **24 hours**?	I did not have diarrhea at all = 0<br>1–2 times = 1<br>3–4 times = 2<br>5 or more times = 3
13. Rate your sense of **smell** in the last **24 hours**	My sense of smell is THE SAME AS usual = 0<br>My sense of smell is LESS THAN usual = 1<br>I have NO sense of smell = 2
14. Rate your sense of **taste** in the last **24 hours**	My sense of taste is THE SAME AS usual = 0<br>My sense of taste is LESS THAN usual = 1<br>I have NO sense of taste = 2
